# Liq_ccRCC: Identification of Clear Cell Renal Cell Carcinoma Based on the Integration of Clinical Liquid Indices

**DOI:** 10.3389/fonc.2020.605769

**Published:** 2021-01-06

**Authors:** Jianhong Zhao, Jiangpeng Wu, Jinyan Wei, Xiaolu Su, Yanjun Chai, Shuyan Li, Zhiping Wang

**Affiliations:** ^1^ Department of Radiology, Lanzhou University Second Hospital, Lanzhou, China; ^2^ Department of Chemistry and Chemical Engineering, Lanzhou University, Lanzhou, China; ^3^ Department of Pathology, Lanzhou University Second Hospital, Lanzhou, China; ^4^ Institute of Urology, Lanzhou University Second Hospital, Key Laboratory of Gansu Province for Urological Diseases, Clinical Center of Gansu Province for Nephrourology, Lanzhou, China

**Keywords:** Liq_ccRCC, clear cell renal cell carcinoma, subtype differentiation, liquid indices, machine learning

## Abstract

Currently, preoperative diagnosis and differentiation of renal clear cell carcinoma and other subtypes remain a serious challenge for doctors. The liquid biopsy technique and artificial intelligence have inspired the pursuit of distinguishing clear cell renal cell carcinoma using clinically available test data. In this work, a method called liq_ccRCC based on the integration of clinical blood and urine indices through machine learning approaches was successfully designed to achieve this goal. Clinically available biochemical blood data and urine indices were collected from 306 patients with renal cell carcinoma. Finally, the integration of 18 top-ranked clinical liquid indices (13 blood samples and 5 urine samples) was proven to be able to distinguish renal clear cell carcinoma from other subtypes of renal carcinoma by cross-valuation with an AUC of 0.9372. The successful introduction of this identification method suggests that subtype differentiation of renal cell carcinoma can be accomplished based on clinical liquid test data, which is noninvasive and easy to perform. It has huge potential to be developed as a promising innovation strategy for preoperative subtype differentiation of renal cell carcinoma with the advantages of convenience and real-time testing. liq_ccRCC is available online for the free test of readers at http://lishuyan.lzu.edu.cn/liq_ccRCC.

## Introduction

Renal cell carcinoma (RCC) is the primary malignant tumor in renal tumors, occupying the sixth place globally with regard to tumor death; it is the second leading cause of death among urinary system tumors only after bladder cancers (1). There are several subtypes of RCC, for which the growth rate, mode, and metastasis rate vary greatly. Among these subtypes, clear cell renal cell carcinoma (ccRCC) is the most prevalent type, accounting for about 75%–80% of all diagnosed instances (2) and 90% of cases that metastasize (3). Approximately 25%–30% of patients with ccRCC present with metastatic disease at the time of diagnosis (4). Therefore, the surgical methods and prognosis for ccRCC exhibit great differences compared with other subtypes. Hence, an accurate preoperative identification of ccRCC will contribute significantly to the success rate of surgery and survival rate of patients. However, CT or MR enhancement, which are currently the most dominant imaging diagnostic methods for renal cancers, have frequently failed to differentiate between different subtypes of RCC; this affects the treatment scheme, surgical approach, and prognosis of patients. Therefore, exploring new methods for simply and quickly distinguishing ccRCC from other subtypes is still a serious challenge for doctors.

Many researchers have contributed to the identification of ccRCC. For example, from the medical imageology standpoint, Dong et al. investigated the contrast-enhanced ultrasound (CEUS) method (5), and Wei et al. analyzed the dual energy spectral CT method (6) with this goal in mind; both achieved good identification performance. Young et al. provided novel evidence that multiphasic multidetector CT may assist in the discrimination of ccRCC from oncocytoma, papillary RCC, and chromophobe RCC (7). However, these methods are radioactive, time-consuming, or complex. Therefore, they have not been widely applied in clinical practice. Wang et al. utilized a 44-gene expression signature from microarray analysis to accurately discriminate ccRCC from different subtypes with an overall accuracy of 95.7% based on 5-fold cross-validation, which was beneficial for accelerating the development of the gene expression profile (8). Recently, with the concept of liquid biopsies continuing to evolve, various biomarkers have been rapidly emerging in the field of diagnosis, prediction, and prognosis of ccRCC. For example, James et al. applied an enhanced RT-PCR technique to test MN/CA9 mRNA that was expressed in the peripheral blood of patients with renal cancer. The results show that 86% of ccRCC had a positive expression of MN/CA9 mRNA, and no patient with a benign renal tumor exhibited MN/CA9 expression (9). Zhao et al. used real-time PCR to measure microRNA miR-210 in serum and found that the average level of miR-210 was significantly higher in ccRCC patients than in controls (*p*<0.001) with an area under the curve (AUC) of 0.874 (10). There is mounting evidence that serum-circulating long noncoding RNAs (lncRNAs) have great potential as practical biomarkers for clinical diagnosis. Wu et al. conducted an adequate investigation of the levels of the 5-lncRNA signature to build a risk model that could distinguish ccRCC samples from healthy controls with an AUC value of 0.9000 (11). In addition, serum histidine and plasma tryptophan were employed to correctly classify 85.5% of control and 84.7% of case samples with the logistic regression model (12). However, this requires extremely high sensitivity in terms of detection technology because of the low levels of these biomarkers that are released into the blood.

Therefore, we construct a simple, effective, and noninvasive method to differentiate ccRCC from other RCC subtypes. In this work, inspired by multi-analyte blood tests, which can reveal greater correlations between complex associations (13), and the success of machine learning in support decision systems (14, 15), we sought to find a link between ccRCC and clinical liquid data, including blood and urine indices that are easy and cost-effective to detect through machine learning approaches.

## Materials and Methods

### Source of Materials

A total of 306 samples that were collected in the Lanzhou University Second Hospital were used to build the model, of which 269 samples were used to train the model and 37 samples were used to test the performance of the model. From among these samples, 240 samples of ccRCC were classified as positive samples, and the remaining were negative samples, including papillary, chromophobe, and other rare renal tumors. Furthermore, all samples were collected with routine blood and urine testing when a patient was first diagnosed with malignant kidney tumors through clinical characteristics and hematological, radiological, and histopathological examinations, etc., by no less than two experienced experts. Each sample consisted of 26 routine blood indices (detected by Sysmex XN9000), 22 blood biochemical indices (detected by Roche COBAS 8000), and 16 numerical routine urine indices (detected by Sysmex UF-1000i). Detailed allocation information about the data sets is shown in **Table 1**, and general information about the indices is listed in **Supplementary Table S1**. The study was approved by the ethics committee of Lanzhou University Second Hospital. Written informed consent was obtained from all participants.

**Table 1:** Detailed division number and general information of the data set of RCC.

	Training set	Testing set	Total
ccRCC	219	21	240
Other subtypes of RCC	50	16	66

### Machine Learning Method

The random forest (RF) method is a flexible and practical classifier among many supervised machine learning algorithms and has been widely used in scientific research and practical applications. The most prominent advantages of the RF algorithm are random sampling and random feature selection, which can ensure the accuracy and stability of a model. In addition, it can reduce the dimension of high-dimensional data and has a strong generalization ability for data sets about which little is known. Moreover, it can monitor the error, strength, and correlation of an out-of-bag set; it can also present the importance of a set’s features through permutation. Generally speaking, the RF method mainly contains two parameters to be adjusted, namely the number of trees (ntree) and the number of randomly selected features to be split at each node (mtry). Because of the unique advantages of RF, this algorithm was used for training and predicting samples in this study.

The research process can be roughly divided into three successive stages. First, all blood and urine indices were used to construct a suitable classification model. The main purpose of this is to obtain the importance ranking of each index for high-performance prediction outcomes. Second, with the adjustment of ntree and mtry parameters, various models with different outcomes were built by increasing the number of important indices one by one based on 10-fold cross-validation. The value of ntree increased from 500 to 2000 with a step size of 100, whereas the value of mtry increased from 2 to 15 with a step size of 1. Third, according to the principle that an appropriate number of top-ranking indices could achieve a comparable prediction performance as using all the indices, the final model was determined by the 20 most relevant indices with ntree and mtry being 1400 and 2, respectively. RF was applied to the RandomForest package of R v4.6-7.

### Validation Method

There are two different yet complementary methods for the model evaluation process, including 10-fold cross-validation of the training set and external verification of the testing set to obtain robust prediction performance for identifying ccRCC. Indeed, 10-fold cross-validation was used to divide the training set into 10 nonoverlapping parts, one of which was used for internal verification, and the remaining parts were used for internal model training. After this process had been repeated 10 times, each sample could be used to test the model once. Therefore, 10-fold cross-validation is a powerful and persuasive method for verifying the prediction ability of a model.

By contrast, external verification of the testing set was only employed to test the model performance; it did not contribute to the training process of the model, which was very different from that of the training set based on 10-fold cross-validation.

## Results

**Figure 1:**
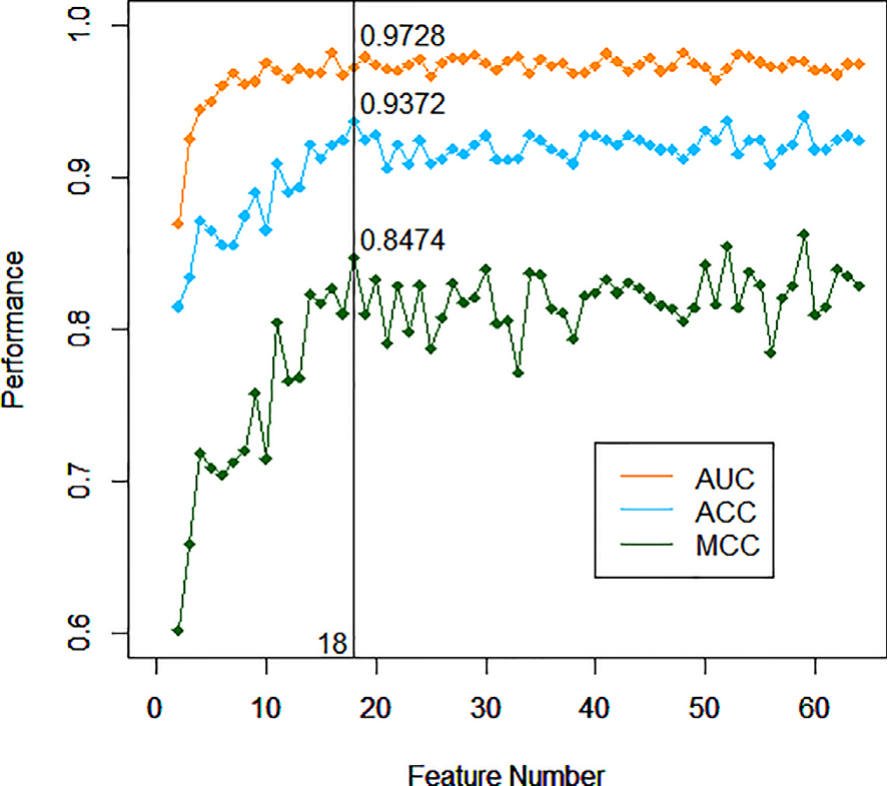
Performance of different models with incremented number of top-ranking indices of the training set.

**Figure 2:**
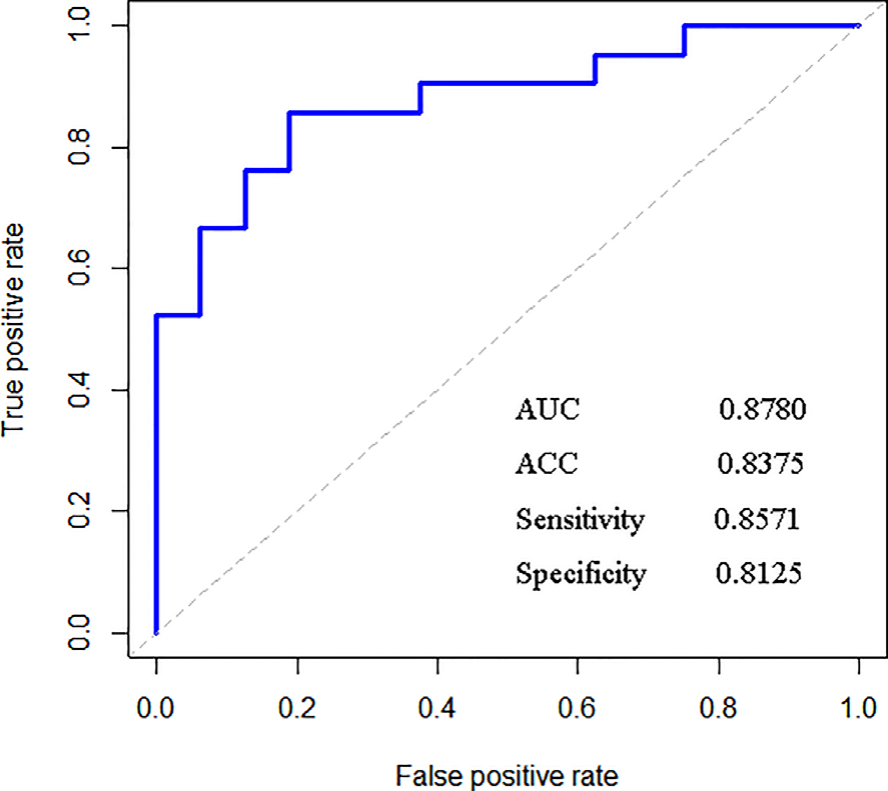
Results of the external verification of the testing set.

After training based on 10-fold cross-validation, a model composed of 18 top-ranking indices selected by the RF method displayed relatively good performance in identifying ccRCC with high sensitivity, specificity, accuracy (ACC), and associated AUC values of 0.9456, 0.9097, 0.9372, and 0.9728, respectively, as shown in **Figure 1**. The specific information of these indices is listed in **Table 2**. Apart from the ability to discriminate ccRCC from various other types of malignant kidney tumors in the training set, the model had satisfactory prediction outcomes for the testing set with an ACC of 0.8375 and AUC of 0.8780 as shown in **Figure 2**. These results indicate that the model formed by the complex combination of 18 routine blood and urine indices exhibited good performance in identifying ccRCC; thus, the model could help patients predict the severity of their disease in advance and avoid unnecessary histopathological examinations.

**Table 2:** Top-ranking indices selected by the RF algorithm listed in order of decreasing importance.

Rank	Feature (Abbreviation)	Reference range
1	Immature granulocytes (IG#)	0.00-0.03 (10^9^/L)
2	Immature granulocyte ratio (IG%)	0.0-0.5 (%)
3	Non-lysed-red blood cells (N-L-RBC#)	
4	Magnesium (Mg)	0.70-1.20 (mmol/L)
5	Red blood cells (RBC)*	0.0-20.0 (/ul)
6	Globulin (GLO)	15.0-35.0 (g/L)
7	ALB/GLB	1.10-2.50
8	Phosphorus (PHOS)	0.80-1.45 (mmol/L)
9	Blood urine nitrogen (BUN)	1.8-8.0 (mmol/L)
10	Platelet (PLT)	100-300 (10^9^/L)
11	Bacteria (BACT)	0.0-1000.0 (/ul)
12	Total protein (TP)	60.0-85.0 (g/L)
13	Albumin (ALB)	35.0-55.0 (g/L)
14	Calcium (Ca)	2.10-2.80 (mmol/L)
15	Mucous strands (MUCUS)	(/ul)
16	Conductivity (Cond.)	3.0-39.0 (mS/cm)
17	Direct bilirubin (DBIL)	0.0-6.8 (μmol/L)
18	Red blood cells (RBC)^#^	4.00-5.50 (10^12^/L)

*RBC from urine examination.

^#^RBC from blood examination.

**Figure 3:**
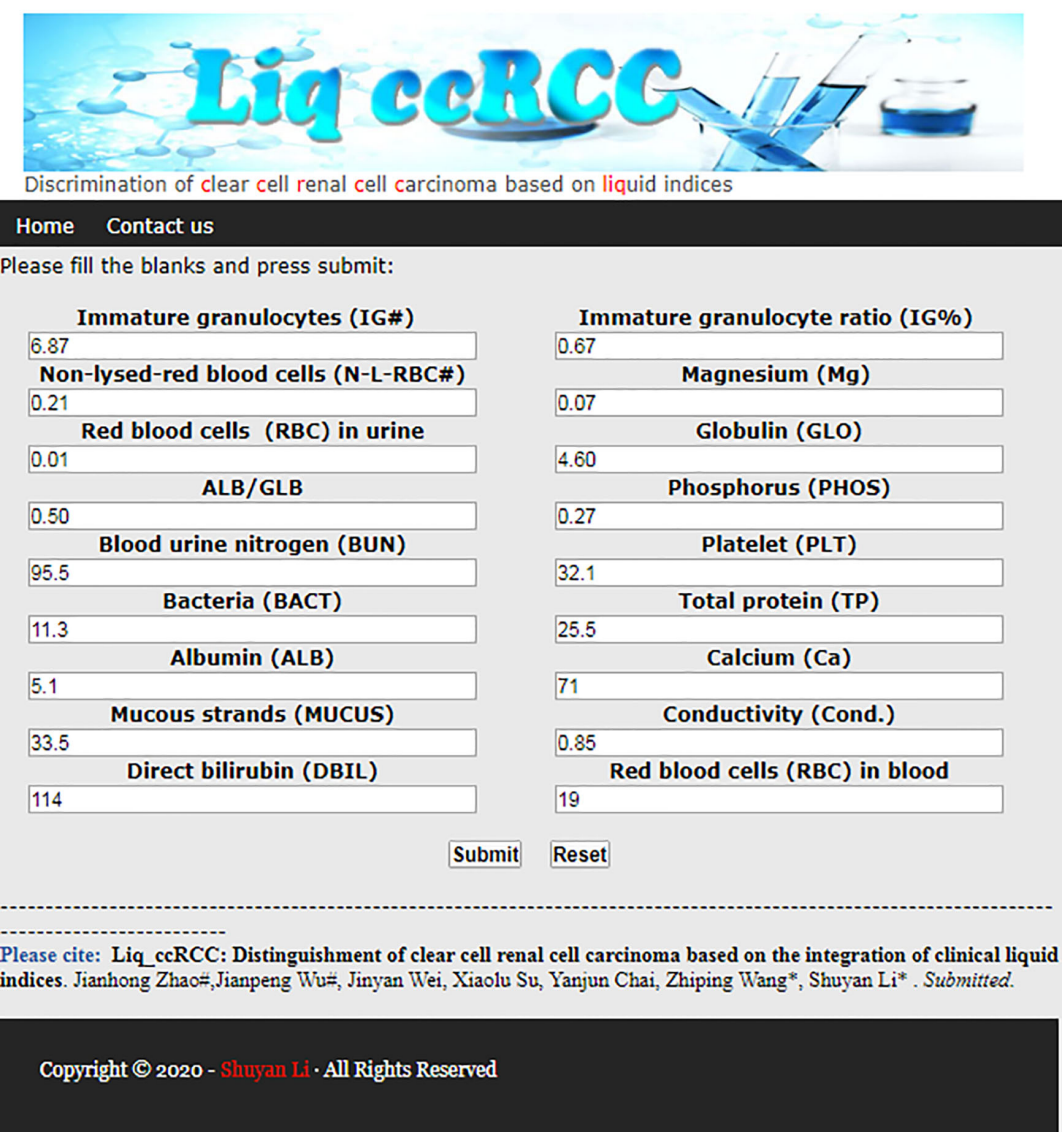
Web server of liq_ccRCC method.

An interactive web server of liq_ccRCC was developed for users to test, explore, and experience this method. It is very convenient and straightforward that users need only enter the related value into the corresponding text box according to the requirements of the interface. After clicking the “Submit” button, the prediction information of the sample is presented in the results interface after calculation and analysis. The main page of this website for ccRCC discrimination is shown in **Figure 3**. This method has the potential to be developed into a promising tool for the discrimination of ccRCC from other types of kidney cancers.

## Discussion

During the training process, the importance of an appropriate number of top-ranking indices should be highlighted in the context of an urgent requirement for high-efficiency solutions and easy-to-perform models. Thus, the 18 top-ranking indices are hand-picked by the RF method based on the principle that this low number of indices can attain comparative prediction ability to the entire set of indices. Although the AUC value of the model did not reach the optimal choice with the combination of 18 indices, both the Matthews correlation coefficient (MCC) and ACC reached their peaks. The difference between the AUC at the time and the nearby AUC values was very small. After comprehensive consideration, 18 indices became the best choice for identifying ccRCC without compromising on performance. Although ccRCC cannot be diagnosed by symptoms alone, the prewarning method can provide warning information to patients for more in-depth examinations. In particular, when the pattern of tumor cells is abnormal or the availability of lesion samples is limited, this auxiliary method is expected to offer a potential future avenue for some special ccRCC diagnosis. The method using the combination of key indices through the RF algorithm has been proven to be stable and reliable based on the results of the training set and the external testing set.

To further prove the generalization ability of this method, another external testing data set with 79 RCC samples, including 61 ccRCC and 18 non-ccRCC, were collected from the First Hospital of Lanzhou University. Routine blood indices were detected by Mindray BC 6800. The blood biochemical indices were detected by BECKMAN, and the routine urine indices were detected by Sysmex UF-1000i. Detailed information about this new data set is listed in **Supplementary Table S2**. The study was approved by the ethics committee of the First Hospital of Lanzhou University. Based on the same method described earlier, the new samples with 18 blood and urine indices showed comparable identification results for ccRCC in terms of sensitivity, specificity, accuracy, and AUC of 0.8229, 0.8000, 0.8222, and 0.8507, respectively. These new samples revealed comparable identification ability with the first testing set using the proposed method although the test data were collected with different testing equipment than the training data set. These results suggest that this method possesses good generalization ability and is able to tolerate systematic errors between different testing instruments to some extent. Therefore, this technology looks promising for differentiating ccRCC from other subtypes of RCC preoperatively in the future.

**Figure 4:**
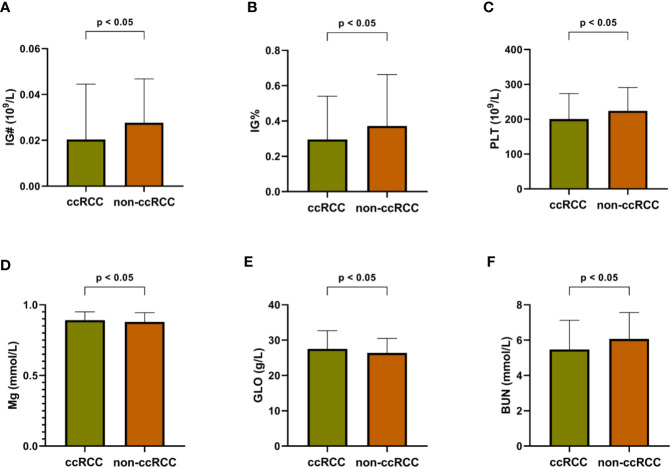
Differentially expressed blood indices between ccRCC and non-ccRCC. **(A)** Immature granulocytes (IG#); **(B)** Immature granulocyte ratio (IG%); **(C)** Platelet (PLT); **(D)** Magnesium (Mg); **(E)** Globulin (GLO); **(F)** Blood urine nitrogen (BUN).

To explore more valuable information, all 18 indices selected from the RF algorithm, including 4 routine blood indices, 9 blood biochemical indices, and 5 numerical routine urine indices were analyzed by Mann–Whitney U tests, for both ccRCC and non-ccRCC samples. Finally, six differentially expressed blood indices were identified as providing some novel pathological insights and potential clinical application opportunities as shown in **Figure 4**. Immature granulocytes (IG#) and immature granulocyte ratio (IG%) are not discussed commonly in terms of clinical relevance for this disease. However, IG% played a special role in the severity of sepsis (16). In **Figures 4A, B**, the expression levels of these two indices for ccRCC were significantly lower than those of non-ccRCC patients; therefore, they may be two useful biomarkers for reflecting the progress of renal tumors. Previous studies report that platelets (PLT) are related to tumor angiogenesis; they promote metastasis and contain diverse angiogenic factors that are associated with various stages of tumor development (17). Thus, the PLT in **Figure 4C** has great potential for enhancing the identification probability of ccRCC. The function of magnesium for genetic instability and promotion of tumorigenesis in a body has received some pertinent attention (18). It is meaningful that there is a significant difference in the expression of magnesium between ccRCC and non-ccRCC samples (**Figure 4D**). As the main serum protein, globulin (GLO) has always been a common and important marker, having a significant impact on the inflammation process (19). The improved GLO can be regarded as an important risk factor for ccRCC, as shown in **Figure 4E**. Urea is a nitrogen-containing metabolite produced during protein metabolism (20). In this study, we found that the urea level of patients with ccRCC was significantly lower than that of other negative samples (**Figure 4F**), which may be an independent predictor of the risk of ccRCC in advance.

Supplementing these differentially expressed blood indices remains an important driving force for promoting a better understanding of the ccRCC process. The random forest method can not only absorb these differentially expressed indices, but also integrate some conventional blood and urine indices into a prewarning system for identifying ccRCC after comprehensive consideration. Although none of the urine indices suggests significant differential expression levels, these parameters play an indispensable part in the nodes of each random tree to help accurately distinguish ccRCC from other types of renal tumors. It is a complex combination of all selected indices that leads to a high-performance ccRCC discrimination system, which implies that no single indicator is fully capable of identifying ccRCC from among complex diseases.

In order to further evaluate the performance of our method, the identification results of ccRCC were compared between this work and recently published works pursuing the same goal. **Table 3** shows that our method can satisfy the enormous demands of discovering ccRCC with increased sensitivity and specificity compared with recently published methods. At the same time, several important blood and urine indices have been developed and applied in the RF algorithm to identify the relationship between ccRCC and other types of renal tumors and to facilitate the rapid and accurate diagnosis of ccRCC.

**Table 3:** Performance comparison of different methods for identifying ccRCC.

Identification method		Sample number	Sens (%)	Spec (%)	AUC	ACC
**This work**		**306**	**94.56**	**90.97**	**0.9372**	**97.28**
Gene expression profiling (8)		295	98.10	NA	NA	95.70
Serum lncRNA signature (11)		51	79.20	88.90	0.9000	84.10
Serum histidine and plasma tryptophan (12)		242	84.70	85.50	0.9160	85.12
Multiphasic multidetector CT (7)	Discrimination of ccRCC from					
	oncocytoma	108	86.00	43.00	NA	77.00
	papillary RCC	119	94.00	62.00	NA	85.00
	chromophobe RCC	97	92.00	25.00	NA	84.00

Collectively, our results suggest that there are strong associations between various types of renal carcinoma, specific hematological characteristics and urine indicators. The diagnostic method liq_ccRCC, which was built based on these key indices in routine clinical settings, appears to be able to accurately differentiate ccRCC, especially with atypical histological presentation. The high-level discriminatory performance between ccRCC and other subtypes of RCC demonstrates that this method has huge potential to be extended and applied for the early warning of other malignant diseases after sufficient cognition by machine learning. More clinical trials are needed to test the reliability and stability of this method. Nonetheless, our results indicate that a new class of ccRCC diagnostic methods may provide significant future value to patients.

## Data Availability Statement

The original contributions presented in the study are included in the article/**Supplementary Materials**. Further inquiries can be directed to the corresponding authors.

## Author Contributions

ZW and SL conceived and designed this work. JZ and JWu constructed the identification model, built the web server and wrote the manuscript. JWe collected the data of blood indices. XS collected the data of urine indices. YC did the validation of the built model and helped to revise the manuscript. All authors contributed to the article and approved the submitted version.

## Funding

This work was supported by the health industry scientific research project of Gansu province (GSWSKY-2015-54).

## Conflict of Interest

The authors declare that the research was conducted in the absence of any commercial or financial relationships that could be construed as a potential conflict of interest.
